# Supplementary material for: WordCommentsAnalyzer: A windows software tool for qualitative research
Source: F1000Res. 2018 Sep 4;7:536. Originally published 2018 May 3. [Version 2] doi: 10.12688/f1000research.14819.2 (PMC6137411; doi:10.12688/f1000research.14819.2)
Supplement: Supplementary file 3 [file f1000research-7-17614-s0002.tgz › b2a79496-4ada-47c4-9682-a99ce867836d.zip!/Eight Iranian Health Journals/Archives of Rehabilitation_20.docx]

© Archives of Rehabilitation. This work is licensed under CC BY-NC 4.0 (https://creativecommons.org/licenses/by-nc/4.0/).

Volume & Issue: دوره 14 - شماره 4

Title: بررسی نیاز‌های توجهی کنترل الگوی تنفس و ارتباط آن با پیامد‌های متابولیکی تنفس در افراد سالم

http://rehabilitationj.uswr.ac.ir/article-1-796-fa.html

هدف: ازآنجاکه الگوی تنفس طبیعی برای تنظیم متابولیسم طبیعی بدن ضروری است، هدف از این تحقیق، مقایسۀ کنترل الگوی تنفس در حالت مواجهه با تکلیف شناختی واکنش و حالت استراحت روی افراد سالم است. روش بررسی: در این تحقیق شبه‌تجربی، ۲۴ فرد سالم، یعنی ۱۴ مرد و ۱۰ زن، به‌صورت ساده و در دسترس انتخاب شدند. داده‌‌های اسپیرومتری شامل زمان‌‌های دم و بازدم و زمان کل یک چرخۀ تنفسی، حجم دم و حجم دقیقه‌ای، تعداد تنفس در دقیقه و نسبت دم به کل چرخۀ تنفس بودند و داده‌‌های کپنوگرافی شامل فشار دی‌اکسیدکربن هوای بازدمی در سطح دهانی در دو وضعیت با تکلیف و بدون تکلیف زمان واکنش دیداری بودند که به‌طور هم‌زمان، از تمام آزمودنی‌ها اخذ شد. داده‌های حاصل با آزمون تی زوجی و تعیین همبستگی تحلیل شد. یافته‌ها: میانگین فشار گاز دی‌اکسیدکربن در هوای بازدم، زمان دم، زمان بازدم و زمان کل سیکل تنفسی در زمان استراحت، به‌صورت معنا‌داری بزرگ‌تر از موقعیت اجرای تکلیف یاد شده بود (۰/۰۵>P). همچنین، هم‌بستگی آماری متوسط معنادار مستقیم، بین تغییرات حجم جاری، تغییرات زمان دم، زمان بازدم، زمان کل چرخۀ تنفسی و تغییرات نسبت دم به کل زمان چرخۀ تنفسی با تغییرات فشار گاز دی‌اکسیدکربن در هوای بازدمی وجود داشت. نتیجه‌گیری: بارِ شناختی، تأثیر بسزایی در تغییر الگوی تنفس در افراد سالم داشته و هم‌بستگی مناسبی بین الگو‌های تنفسی و پیامد‌های متابولیکی تنفس در افراد سالم وجود دارد.

Title: تأثیر یک دورۀ برنامۀ حرکات اصلاحی بر تعادل پویا در افراد مبتلا به اسکولیوز ایدیوپاتیک

http://rehabilitationj.uswr.ac.ir/article-1-1218-fa.html

هدف: هدف از پژوهش حاضر، بررسی تأثیر یک دورۀ برنامۀ حرکات اصلاحی بر تعادل پویا در افراد مبتلا به اسکولیوز ایدیوپاتیک بود. روش بررسی: برای اجرای این پژوهش، ۲۰ فرد مبتلا به بیماری اسکولیوز ایدیوپاتیک با میانگین سن ۲/۷±۱۳/۶ سال‌ و قد ۱۳/۹±۱۵۳/۳ سانتی‌متر و وزن ۹/۶±۴۴/۴ کیلوگرم، به‌صورت داوطلب در تحقیق شرکت کردند و به‌طور تصادفی، به دو گروه تجربی (۱۰=N) و گروه کنترل (۱۰=N) تقسیم شدند. ابتدا، محقق برای آزمودنی‌ها، مراحل و نحوۀ برگزاری آزمون‌ها و برنامۀ تمرین را توضیح داد. سپس، قد و وزن آزمودنی‌ها اندازه‌گیری شد. درنهایت، آزمون‌ها به این‌صورت گرفته شد: از حالت نشسته روی تکیه‌گاه بلندشدن و راه‌رفتن به‌صورت متوالی با پاشنه‌پنجه، برای سنجش تعادل پویا. درنهایت، گروه تجربی مدت ۴ هفته، هفته‌ای ۳ جلسه و هر جلسه، ۴۵ دقیقه برنامۀ تمرین اصلاحی را انجام دادند و گروه کنترل هیچ تمرینی نکردند. بعد از ۴ هفته، هر دو گروه دوباره آزمون‌های فوق را انجام دادند. در پایان، برای تجزیه‌وتحلیل داده‌ها از آزمون آماری تی مستقل و تی هم‌بسته استفاده شد. یافته‌ها: نتایج نشان داد یک دورۀ تمرین حرکات اصلاحی در آزمون، بر متغیرهای طول گام (۰/۰۱۲=P) و انتهای حرکت (۰/۰۳۰=P) تأثیر معناداری دارد؛ اما، بر متغیر سرعت (۰/۰۸۳=P) تأثیر معناداری ندارد. همچنین، بین دو گروه در دو متغیر طول گام (۰٫۰۲=P) و انتهای حرکت (۰/۰۱۰=P) اختلاف معناداری وجود دارد؛ اما، در متغیر سرعت گام (۰/۱۴۱=P) اختلاف معناداری وجود ندارد. در آزمون راه‌رفتن به‌صورت متوالی با پاشنه‌پنجه نیز بر متغیر سرعت راه‌رفتن (۰/۰۰۱=P) تأثیر معنادار است؛ اما بر متغیرهای انتقال وزن (۰/۰۱۲=P) و شاخص بلندشدن (۰/۹۵=P) تأثیر معنادار نیست. در مقایسۀ دو گروه، در متغیر سرعت راه‌رفتن (۰/۰۰۰=P) اختلاف معناداری وجود دارد و در متغیرهای انتقال وزن (۰/۰۱۰=P) و شاخص بلندشدن (۰/۷۳=P) اختلاف معناداری وجود ندارد.  نتیجه‌گیری: باتوجه‌به نتایج، می‌توان گفت یک دورۀ تمرین حرکات اصلاحی برای بهبود بیماری اسکولیوز ایدیوپاتیک مناسب است.

Title: بررسی اثربخشی ترکیب دو فن تصویرسازی ذهنی و ماساژدرمانی بر میزان خشم کودکان مبتلا به سرطان

http://rehabilitationj.uswr.ac.ir/article-1-1268-fa.html

هدف: خشم و پرخاشگری از عوارض بیماری‌های مزمن، ازجمله سرطان، در کودکان بیمار به‌شمار می‌آید. هدف از این مطالعه بررسی اثربخشی تلفیق دو فن تصویرسازی ذهنی و ماساژدرمانی بر میزان خشم کودکان مبتلا به سرطان است. روش بررسی: این مطالعۀ مداخله‌ای از نوع کارآزمایی بالینی بود و در کلینیک تخصصی ساختمان ارم وابسته به بیمارستان بوعلی اردبیل انجام شده است. تعداد ۳۰ کودک مبتلا به سرطان خون و لنفوم با استفاده از روش نمونه‌گیریِ دردسترس انتخاب شدند و به‌طور تصادفی، در دو گروه ۱۵نفری آزمایش و کنترل قرار گرفتند. برای گردآوری اطلاعات، از مقیاس ابراز خشم استیل و همکاران در دو مرحلۀ قبل از کاربست تجربی و بعد از آن، استفاده شد. پس از ارائۀ آموزش‌های لازم به گروه آزمایش، درمانگر روش درمانی را بر هریک از بیماران گروه آزمایش به‌مدت سه هفته و در شش جلسه، یعنی هفته‌ای دو جلسۀ ۲۰دقیقه‌ای اِعمال کرد. برای تجزیه‌وتحلیل داده‌ها، از روش آماری تحلیل پراکنش چندمتغیره استفاده شد. یافته‌ها: نتایج نشان داد که در گروه آزمایش، ازنظر میزان متغیرهای صفت خشم، ابراز خشم ، خشم درونی و کنترل خشم در قبل و بعد از مداخله، تفاوت آماری معناداری دیده می‌شود (۰/۰۰۱>P و ۰/۰۵>P)؛ به این معنی که در گروه آزمایش، میزان صفت خشم، ابراز خشم و خشم درونی کاهش و میزان کنترل خشم افزایش یافته بود؛ اما در گروه کنترل، میزان متغیرهای صفت خشم، ابراز خشم ، خشم درونی و کنترل خشم در قبل و بعد از مداخله، تفاوت آماری معناداری وجود نداشت. نتیجه‌گیری: ترکیب دو روش ماساژدرمانی و تصویرسازی ذهنی در جریان درمان کودکان مبتلا به سرطان می‌تواند موجب کاهش تمایل به پرخاشگری و احساس خشم شود. بنابراین، به‌منظور کاستن از عوارض روان‌شناختی سرطان و درمان‌های مختلف آن، آموزش این دو روش به مسئولان درمان توصیه می‌شود و استفاده از آن‌ها می‌تواند مؤثر واقع شود.

Title: بررسی پیامدها و بازگشت به بازی در فوتبالیست‌های دارای رباط صلیبی قدامی بازسازی‌شده درطول یک سال بعد از جراحی

http://rehabilitationj.uswr.ac.ir/article-1-1091-fa.html

هدف: هدف از پژوهش حاضر، بررسی پیامدها و میزان بازگشت به بازی در فوتبالیست‌های دارای رباط صلیبی قدامی (ACL) بازسازی‌شده درطول یک سال است. روش بررسی: سی‌ودو نفر فوتبالیست که عمل بازسازی ACLداشتند، ازطریق (BPTBG) Bony-Patellar Tendon- Bony graft (تکنیک Modified Clancy) انتخاب و به‌صورت تصادفی، در دو گروه شانزده‌نفریِ بازتوانی تسریعی و سُنتی تقسیم‌بندی شدند. گروه تسریعی، برنامۀ بازتوانی تسریعی را به‌مدت ۲۴ هفته و گروه سُنتی، برنامۀ بازتوانی سنتی را به‌مدت ۳۶ هفته اجرا کردند. در پایان ماه‌های ۴، ۶، ۹ و ۱۲، عملکرد عضلانی عضلات زانو و عملکرد ذهنی و میزان بازگشت به سطوح رقابتی ورزش‌کاران ارزیابی شد. از تحلیل پراکنش با اندازه‌گیری مکرر و آزمون تی برای تجزیه‌وتحلیل نتایج در سطح ۰/۰۵ بهره گرفته شد. یافته‌ها: در هر دو گروه، عملکرد عضلانی اندام جراحی‌شده کمتر از اندام سالم بود. عملکرد عضلانی زانوی جراحی‌شده در گروه تسریعی نسبت‌به گروه سُنتی بهتر بود (P<0/05). عملکرد ذهنی در گروه تسریعی در هر دو مقیاس (لِی ‌شهُلم و سینسیناتی) بیشتر از گروه سُنتی بود (P&le0/05). گروه تسریعی، به سطوح  ۳ و ۴ رقابتی بازگشتند؛ درحالی‌که گروه سُنتی به‌طور معناداری پایین‌تر بود (۱، ۲)، (P&le0/02). نتیجه‌گیری: نتایج نشان داد که نقص در قدرت با نقص در عملکرد ورزشی فوتبالیست‌ها رابطۀ قوی دارد و باعث نقصان فرد در عملکرد ذهنی می‌شود. همچنین برنامۀ تمرین‌های تسریعی (عصبی‌عضلانی) در بازگشت مطمئن‌تر فوتبالیست‌ها به مسابقات نقش کلیدی ایفا می‌کند.

Title: طراحی ابزار روا و پایا جهت ارزیابی صلاحیت بالینی کاردرمانگران

http://rehabilitationj.uswr.ac.ir/article-1-1250-fa.html

هدف: این پژوهش با هدف طراحی و تعیین روایی و پایایی ابزار ارزیابی صلاحیت بالینی کاردرمانگران انجام شد. روش بررسی: در این مطالعه که از نوع ابزارسازی است، با مرور مطالب و چک‌لیست‌ها، تعداد زیادی گویه‌های مربوط‌به صلاحیت بالینی استخراج شد. این گویه‌ها در دو جلسۀ گروهی و زیر نظر پانزده تن از خبرگان مطرح و تحلیل شد و درنهایت، پرسش‌نامه‌ای با ۶۶ گویه تهیه شد. سپس، براساس نظر متخصصان کاردرمانی و شیوۀ لاواشه، روایی صوری و محتوایی پرسش‌نامه به دو روش کمّی و کیفی تعیین گردید. به‌منظور تعیین پایایی از روش مطالعۀ آزمایشی، آزمون‌بازآزمون و محاسبۀ هم‌بستگی درونی برای تأیید پایایی استفاده شد. یافته‌ها: ابزار ارزیابی صلاحیت بالینی با ۵۴ پرسش در ۷ حیطه طراحی شد که عبارت است از: تفکر انتقادی، فرایند عملکرد، ارتباط مؤثر، مسئولیت حرفه‌ای، مدیریت محیط درمان، دانش عملکردی و اقدام به رشد حرفه‌ای. روایی صوری و محتوایی با نظر هیئت خبرگان محاسبه و با CVR=۰/۸۴ تأیید شد. پایایی ابزار تهیه‌شده در مطالعۀ آزمایشی نیز بررسی و روش آزمون - بازآزمون، ضریب هم‌بستگی پیرسون ۰/۹۹۵ را نشان داد. هم‌بستگی درونی پرسش‌ها نیز با محاسبۀ ضریب آلفای کرونباخ بررسی شد و عدد ۰/۹۷۳ را نشان داد. نتیجه‌گیری: ابزار مناسب برای بررسی صلاحیت بالینی کاردرمانگران با ۵۴ گویه در ۷ حیطه تهیه شد. روایی و پایایی این ابزار را خبرگان و نمونۀ ۳۰‌نفرۀ کاردرمانگران تأیید کردند.

Title: بررسی ضخامت اولتراسونیک عضلات دیوارۀ طرفی شکم حین انقباض عضلات کف لگن در زنان مبتلا به بی‌اختیاری استرسی ادرار با کمردرد مزمن و بدون کمردرد مزمن

http://rehabilitationj.uswr.ac.ir/article-1-1138-fa.html

هدف: بی‌اختیاری ادراری از اختلالات مجاری ادراری تحتانی و حاصل کاهش فعالیت عضلات کف لگن است. این اختلال باتوجه‌به فعالیت هم‌زمان عضلات کف لگن و دیوارۀ شکم برای کنترل عمل دفع و برقراری ثبات ناحیۀ کمری‌لگنی رخ می‌دهد. این مطالعه با هدف بررسی تأثیر فعالیت عضلات کف لگن بر ضخامت اولتراسونیک عضلات دیوارۀ طرفی شکم در زنان مبتلا به بی‌اختیاری استرسی ادرار با کمردرد مزمن و بدون کمردرد مزمن انجام شد. روش بررسی: مطالعه دربارۀ هجده زن مبتلا به بی‌اختیاری استرسی ادراری با کمردرد مزمن (نُه نفر) و بدون کمردرد مزمن (نُه نفر) و ده زن سالم انجام شد. پس از ثبت اطلاعات دموگرافیک و ارزیابی عملکرد عضلات کف لگن، ضخامت اولتراسونیک عضلات دیوارۀ طرفی شکم حین استراحت و انقباض عضلات کف لگن اندازه گیری شد. آزمون‌های کولمگروف اسمیرنوف، آنالیز پراکنش یک‌طرفه، کروسکال والیس و پیرسون برای تجزیه‌وتحلیل داده‌ها استفاده و مقادیر P<0/05 به‌عنوان سطح معنادار درنظر گرفته شد. یافته‌ها: ضخامت عضلات دیوارۀ طرفی شکم در حالت استراحت عضلات کف لگن بین سه گروه، اختلاف معنا‌داری نداشت (P<0/05)، ولی حین حین انقباض عضلات کف لگن، ضخامت عضله عرضی شکم تفاوت معناداری  نشان داد (P=0/003). همچنین اختلاف سطح عملکرد و مقادیر فشار داخل واژه حین انقباض عضلات کف لگن، بین سه گروه معنادار بود (P=0/001). نتیجه‌گیری: تغییر ضخامت اولتراسونیک عضلۀ عرضی شکم حین انقباض عضلات کف لگن در زنان سالم، نشان‌دهندۀ برهم‌خوردن الگوی فعالیت هم‌زمان عضلات دیوارۀ طرفی شکم وکف لگن در زنان مبتلا به بی‌اختیاری استرسی ادراری با کمردرد مزمن و بدون کمردرد مزمن است.

Title: مقایسۀ سواد خواندن دانش‌آموزان مبتلا به آسیب بینایی و دانش‌آموزان با بینایی هنجار

http://rehabilitationj.uswr.ac.ir/article-1-1113-fa.html

هدف: اگر خواندن و نوشتن و حساب‌کردن را سه مهارت اصلی آموزش در دورۀ ابتدایی بدانیم، خواندن، سنگ زیربنای دو مهارت دیگر است؛ ازاین‌رو، اطلاع‌یافتن از فرایند خواندن و روش‌های آن از مسائل لازم است. هدف از اجرای این پژوهش، بررسی پیشرفت خواندن در دانش‌آموزان مبتلا به آسیب بینایی پایۀ چهارم ابتدایی و مقایسۀ آن‌ها با دانش‌آموزان هم‌پایه با بینایی هنجار بود. روش بررسی: پژوهش حاضر از نوع مقایسه‌ای و با استفاده از آزمون دفترچه‌های استاندارد‌شدۀ بین‌المللی پرلز (۲۰۰۶) انجام شد. ۲۷ نفر از دانش‌آموزان پایۀ چهارم ابتدایی در مدارس استثنایی شهرهای تهران و ری و قم به‌روش جامعۀ آماری دردسترس انتخاب شدند. سپس محققان، مقایسه‌ای برقرار کردند بین این دانش‌آموزان با همان تعداد دانش‌آموز با بینایی هنجار از مدارس مجاور که به شکل تصادفی ساده انتخاب شدند. برای تجزیه‌وتحلیل داده‌ها نیز از روش آماری تی مستقل استفاده شد.  یافته‌ها: نتایج نشان داد که در سواد خواندن (۰/۰۱۷=P) و درک متون ادبی بین دانش‌آموزان آسیب‌دیدۀ بینایی و دانش‌آموزان با بینایی هنجار تفاوت معنا‌دار وجود دارد (۰/۰۰۷=P)؛ درحالی‌که در درک متون اطلاعاتی تفاوت معنا‌داری بین دانش‌آموزان مبتلا به آسیب بینایی با دانش‌آموزان با بینایی هنجار دیده نشد (۰/۰۵۳=P). نتیجه‌گیری: آسیب بینایی بر میزان سواد خواندن مؤثر است؛ بنابراین نیاز به توجه ویژه و بازنگری در برنامۀ آموزش خواندن مدارس ویژۀ دانش‌آموزان مبتلا به آسیب بینایی ضروری است. توصیه می‌شود معلمان و مسئولان منابع بیشتری با خط بریل، برای کودکان نابینا فراهم کرده و از روش‌های نوین و تقویتی برای پیشرفت سواد خواندن کودکان استفاده کنند.

Title: مقایسۀ تأثیر فوری سه کفی فانکشنال و UCBL و UCBL تغییریافته بر تعادل پویا در افراد مبتلا به صافی انعطاف‌پذیر کف پا

http://rehabilitationj.uswr.ac.ir/article-1-1288-fa.html

هدف: تحقیقات نشان می‌‌دهد کاهش یا افتادگی قوس طولی داخلی پا، یعنی صافی کف پا، می‌‌تواند احتمال آسیب‌دیدن پا را حین فعالیت‌های روزانه نظیر راه‌رفتن افزایش دهد. براین‌اساس، امروزه ارتزهای پا برای کنترل صافی کف پا و جلوگیری از چنین آسیب‌هایی، به‌طور شایع تجویز می‌شود. البته سازوکار تأثیرگذاری این وسایل بر تعادل فرد، کاملاً مشخص نیست و نیازمند استفاده از ابزارهای گوناگون برای کمّی‌سازی مشاهدات بالینی است. هدف: هدف اصلی این مطالعه، بررسی و مقایسۀ تأثیر فوری سه کفی فانکشنال و UCBL و UCBL تغییریافته بر تعادل پویا در افراد مبتلا به صافی انعطاف‌پذیر کف پا است. روش بررسی: بیست مرد و زن جوان مبتلا به صافی انعطاف‌پذیر کف پا (سن ۲/۸ و ۲۳/۵) تحت‌مطالعه قرار گرفتند. آزمودنی‌ها به روش نمونه‌گیری غیراحتمالی و از نمونه‌های دردسترس، برای مطالعه انتخاب شدند. آزمودنی‌ها آزمایش تعادل را در چهار حالت انجام دادند: با کفش به‌تنهایی، با کفش وکفی فانکشنال، با کفش و کفی UCBL و با کفش و کفی UCBL تغییریافته روی دستگاه تعادل بایودکس. در هریک از این حالت‌ها، متغیر نوسانات کلی و داخلی‌خارجی و قدامی‌خلفی اندازه‌گیری شد. آزمون آنالیز اندازه‌های تکراری به‌منظور آنالیز داده‌ها استفاده شد. یافته‌ها: در این مطالعه، نشان داده شد میزان نوسانات کلی در کفی‌های فانکشنال و UCBL به‌طور معناداری، کمتر از UCBL تغییر کرده بود (۰/۰۵>P)؛ اما نوسانات در راستای داخلی‌خارجی و قدامی‌خلفی، در بین سه ارتز تفاوت معنا‌داری نداشت. در مقایسۀ بین کفش با هریک از کفی‌ها، نتایج، معنا‌دارنبودن شاخص‌های ثبات را در هریک از راستاهای داخلی‌خارجی، قدامی‌خلفی و کلی، میان کفش و کفی‌ها نشان داد. نتیجه‌گیری: باتوجه‌ به کاهش معنا‌دار نوسانات پاسچرال در کفی‌های‌ UCBL و فانکشنال نسبت‌به کفی UCBL تغییریافته، به‌نظر می‌رسد تفاوت ساختاری ارتزها بر بهبود تعادل در افراد مبتلا به صافی کف پای انعطاف‌پذیر مؤثر باشد. معنا‌دارنشدن شاخص‌های تعادلی میان کفش و کفی‌ها بیانگر این بود که کفش به‌تنهایی نیز بر کاهش نوسانات پاسچرال مؤثر بوده است؛ بنابراین، به‌دلیل کارایی مؤثر کفش، کفی‌ها بدون دورۀ انطباقی نتوانستند بر ظرفیت تعادلی افراد مؤثر باشند.

Title: کیفیت زندگی بیماران مبتلا به کمردرد غیراختصاصی مزمن

http://rehabilitationj.uswr.ac.ir/article-1-1170-fa.html

هدف: کیفیت زندگی مفهومی چندبعدی است و فراتر از بررسی صِرف سلامت جسمانی است. کمردرد از شایع‌ترین اختلالات عضلانی‌اسکلتی است. بیمارهای مزمن مثل کمردرد غیراختصاصی مزمن پیامد‌های مهمی دارند؛ همچون تحول کیفیت زندگی افراد. این مطالعه به‌منظور ارزیابی کیفیت زندگی افراد مبتلا به کمردرد غیراختصاصی مزمن اجرا شد. روش بررسی: مطالعۀ حاضر مطالعه‌ای توصیفی است و به‌صورت مقطعی در بیمارستان شهیدبهشتی بابل انجام شده است. حجم نمونه ۱۲۵ نفر برآورد شد و افراد با استفاده از چهار پرسش‌نامۀ اطلاعات دموگرافیک، ارزیابی سطح ناتوانی وکیفیت زندگی و ارزیابی شدت درد ارزیابی شدند. یافته‌ها: شرکت‌کنندگان دامنۀ سنی  18 تا 58 سال داشتند که میانگین و انحراف معیار شدت درد و ناتوانی آن‌ها به‌ترتیب، با 32/21±73/54 و 69/16±56/31 برابر بود. ازنظر جنسیتی و وضعیت تأهل، دربارۀ متغیرهای سلامت جسمانی، سلامت روانی، روابط اجتماعی و سلامت محیط تفاوت معناداری وجود نداشت؛ اما افراد با تحصیلات دانشگاهی نسبت‌به دیگر رده‌های تحصیلی، دربارۀ تمام متغیرهای فوق اختلاف معناداری داشتند. شدت درد با سلامت عمومی و سلامت روان، رابطۀ معکوس و با نمرۀ کلی ناتوانی ارتباط مستقیم دارد. نمرۀ کلی ناتوانی نیز با کیفیت زندگی عمومی، سلامت عمومی، سلامت جسم، سلامت روان و روابط اجتماعی، ارتباط معکوس و با شدت درد رابطۀ مستقیم دارد.  نتیجه‌گیری: باتوجه‌به اینکه شدت درد و ناتوانی غیر از بُعد جسمی، بر سایر ابعاد نیز تأثیر می‌‌گذارد، لازم است در آینده، مداخلاتی نه فقط با تأکید بر جسم، بلکه با تأکید بر بهبودی مسائل روانی و اجتماعی نیز برای این بیماران انجام شود.

Title: تبیین معضلات اخلاقی کاردرمانگران در حیطۀ سلامت روان

http://rehabilitationj.uswr.ac.ir/article-1-1290-fa.html

هدف: کاردرمانگران شاغل در حوزۀ سلامت روان، نسبت‌به سایر حوزه‌ها مشکلات بیشتری را تجربه می‌کنند. هدف از این مطالعه، تبیین تجربیات کاردرمانگرانی است که با مسائل اخلاقی در توان‌بخشی روانی‌اجتماعی مواجه هستند.  روش بررسی: این مطالعه از نوع کیفی بود و به شیوۀ تحلیل محتوا انجام شد. نمونه‌ای هدف‌دار متشکل از بیست کاردرمانگر شاغل در بیمارستان‌های روان‌پزشکی انتخاب شدند که داوطلب مشارکت در تحقیق بودند. طیف سِنی افراد تحت‌مطالعه ۲۴تا۴۵ (میانگین ۳۴ سال) و کمترین سابقۀ کاری، یک سال و بیشترینِ آن، بیست سال بود. افراد، درطول پنج جلسه، ازطریق مصاحبۀ متمرکز گروهی، مشکلات مهم تجربه‌شدۀ خود را بیان کردند. سپس معضلات، باتوجه‌به هدف مطالعه، استخراج شد. یافته‌ها: مهم‌ترین مشکلِ مربوط‌به رعایت ضوابط اخلاقی درزمینۀ کار با بیمار روانی، کافی‌نبودن دانش کاردرمانگران برای ارتباط با بیمار و به‌کارگیری خدمات درمانی مناسب بود. کدهای استخراجی از مطالعۀ حاضر، در چهار طبقه قرار گرفت: تناسب درمان با ویژگی‌های بیمار، رفاه بیماران و کیفیت خدمات، تناسب برنامه‌ها با وضعیت بیماران و انتخاب بیماران.  نتیجه‌گیری: نتایج نشان می‌دهد که در حوزۀ سلامت روان، کاردرمانگران با مشکلات متعدد مربوط‌به رعایت اصول اخلاقی مواجه‌اند و برای حل‌وفصل آن‌ها نیازمند ارتقای آگاهی خود در زمینه‌های گوناگون هستند. همچنین، آشکارسازی این مسائل و درمیان‌گذاشتن آن با سایر متخصصان، به‌منظور یافتن راهکارهای موفق برای بهینه‌سازی توان‌بخشی بیماران روانی، ضروری است.

Title: آنالیز نمودار گشتاور زاویۀ مَفصل مچ پا در سرعت‌ها و مراحل مختلف راه‌رفتن جهت طراحی پروتز مچ‌پنجه

http://rehabilitationj.uswr.ac.ir/article-1-1277-fa.html

هدف: هدف این تحقیق آنالیز نمودار گشتاور‌زاویۀ مَفصل مچ پا و تعیین مشخصات مَفصل مچ در مراحل و سرعت‌های مختلف راه‌رفتن برای کاربرد در طراحی پروتزهای مچ‌ و پنجۀ پا بود. روش بررسی: مطالعه به‌صورت مقطعی‌تحلیلی بود و ۲۰ نمونۀ در دسترس انتخاب شدند. آنالیز راه‌رفتن در سرعت‌های مختلف و با دو صفحۀ نیرو و پنج دوربین انجام شد. براساس نمودار گشتاورزاویه، فاز ایستایی به سه مرحله تقسیم شد. مشخصات مَفصل مچ شامل شبه‌سفتی و کار در این مراحل محاسبه شد. مقایسۀ میانگین متغیرها در مراحل و سرعت‌های مختلف راه‌رفتن با آزمون آنالیز پراکنش سنجش مکرر صورت گرفت و برای برازش مدل خطی به داده‌ها، از آنالیز رگرسیون خطی استفاده شد. یافته‌ها: نمودار گشتاور‌زاویه در سرعت‌های آهسته‌تر، در جهت عقربه‌های ساعت و با افزایش سرعت، خلاف جهت عقربه‌های ساعت بود. بهترین خط برازش‌شده به نمودار کار، سرعت راه‌رفتن ۰/۸۱=R2 بود. بین شبه‌سفتی مَفصل مچ در سرعت تند و طبیعی با شبه‌سفتی در سرعت‌های کمتر، تفاوت معناداری وجود داشت (۰/۰۰۱>P). نتیجه‌گیری: رفتار نمودار گشتاور‌ زاویه در سرعت‌های آهسته‌تر نشان‌دهندۀ عملکرد غیرفعال و اتلافی در مَفصل مچ و با افزایش سرعت، نشان‌دهندۀ عملکرد فعال این مَفصل بود. براساس نتایج، مشخصات مَفصل مچ در سرعت‌ها و مراحل مختلف راه‌رفتن تغییر می‌کند. بنابراین، در سرعت‌های پایین‌تر، یک مدل فنر‌دمپر با اتلاف تنظیم‌شده توانایی شبیه‌سازی عملکرد مفصل مچ را دارد؛ گرچه در سرعت‌های بیشتر، باید برای عناصر فعال، سهمی درنظر گرفت.

Title: بررسی کمّی تغییرپذیری دامنۀ حرکتی مفصل آرنج براثر خستگی عضلانی

http://rehabilitationj.uswr.ac.ir/article-1-1057-fa.html

هدف: هدف از این مطالعه بررسی کمّی تغییرپذیری دامنۀ حرکتی مفصل آرنج براثر خستگی اسکلتی‌عضلانی است.  روش بررسی: با نمونه‌گیری غیراحتمالی قضاوتی، تعداد ۱۰ فرد سالم وارد مطالعه شدند. هر آزمودنی چهار فعالیت خسته‌کننده را با دو وزنه که وزنشان ۱۰ و ۲۰ درصد حداکثر توانایی دینامیک فرد در خم‌کردن آرنج سمت غیرغالب بود، در دو سرعت تکرار انجام می‌داد. با استفاده از الکتروگونیامتر و الکترود‌های سطحی الکترومیوگرافی، دامنۀ حرکتی آرنج و فعالیت عضلات بازویی زند اعلایی (براکیورادیالیس) و دوسر بازویی ثبت شد. آنالیز افت‌وخیز بدون روند (DFA) نیز برای محاسبۀ هم‌بستگی تغییرات دامنۀ حرکتی به‌کار رفت. همچنین، تأثیر شرایط آزمایش با آنالیز کروسکال-والیس آزموده شد. یافته‌ها: آنالیز آماری نشان داد که در سه حالت از چهار فعالیت درحال آزمایش، هم‌بستگی بلندمدت بین نوسانات دامنۀ حرکتی وجود داشت (0.5>&alpha&le1). باوجود تفاوت بیومکانیکی بین این سه حالت آزمون، ضریب هم‌بستگی بلندمدت یکسان بود (۰/۵۴۸=P). فعالیت در وضعیت بار زیاد و سرعت تکرار کم، با دیگر وضعیت‌ها متفاوت بود (۰/۰۴۷=P). نتیجه‌گیری: در تمرینات تکرارشوندۀ ورزشی یا توان‌بخشی، پیشنهاد می‌شود بار زیاد با سرعت کم در وضعیت بیومکانیکی کنترل‌شد‌ه‌ای حمل شود؛ زیرا چنین فعالیت‌هایی، در حین خستگی ممکن است به آسیب‌‌های اسکلتی‌‌عضلانی منجر شود.

Title: مقایسۀ تعادل ایستای کودکان نابینا و ناشنوا و همتایان عادی در شرایط مختلف

http://rehabilitationj.uswr.ac.ir/article-1-1292-fa.html

هدف: سیستم‌‌های حسی که شامل دستگاه وستیبولار و حس بینایی و احساس حسی‌پیکری است، نقش مهمی در کنترل حرکتی ایفا می‌کنند. فقدان اطلاعات حاصل از هریک از این سه سیستم می‌تواند روی نوسان بی‌اختیار قامت در وضعیت ایستاده تأثیر بگذارد. روش بررسی: در این پژوهش مقطعی‌مقایسه‌ای، ۱۷ کودک نابینای مادرزادی (۹ دختر و ۸ پسر) و ۳۰ کودک ناشنوا (۱۴ دختر و ۱۶ پسر) ۶تا۱۲‌سالۀ شهر اردبیل، براساس معیار‌های ورود و خروج انتخاب شدند و ۶۰ کودک سالم (۳۰ دختر و ۳۰ پسر) نیز به‌عنوان گروه کنترل، به‌صورت داوطلبانه شرکت کردند. تعادل ایستا با آزمون ایستادن روی یک پا و ایستادن تاندم در وضعیت‌‌های چشم باز و بسته با پایایی (۰/۹۹ تا ۰/۸۷=r) ارزیابی شد. آزمون آماری تحلیل واریانس یک‌طرفه و آزمون تعقیبی LSD برای مقایسۀ بین گروه‌ها استفاده گردید. یافته‌ها: نتایج نشان داد که در تکلیف‌‌های تعادلی تحت مطالعه، عملکرد دختران نابینا و ناشنوا و سالم تفاوت معناداری ندارد؛ درحالی‌که عملکرد پسران ناشنوا و سالم در‌مقایسه‌با پسران نابینا، در همۀ تکلیف‌‌های تعادلی (۰/۰۵>P) غیر‌از ایستادن تاندم با چشم بسته بهتر بود (۰/۵۰۷=P). نتیجه‌گیری: با‌توجه‌به یافته‌‌های طرح حاضر، کودکان نابینا در‌مقایسه‌با کودکان ناشنوا و سالم تعادل ایستای ضعیف‌تری دارند. ازآنجاکه افراد نابینا نمی‌توانند فقدان درون‌داد بینایی برای حفظ ثبات پاسچرال را جبران کنند، کاهش ثبات پاسچرال در وضعیت استاتیک را نشان می‌دهند. بنابراین، شناسایی جامع عوامل مؤثر بر تعادل و مشکلات و ضعف تعادلی در زمان مناسب، توجه به این عوامل در برنامه‌‌های تمرینی و تقویت آن در کودکان با معلولیت حسی به‌خصوص نابینایان، استفاده از نیرو‌های متخصص و نیز توجه ویژه به فعالیت بدنی این کودکان در مدارس پیشنهاد می‌شود.

Title: توسعه و تست یک عینک واسط انسان با کامپیوتر مبتنی‌بر سیگنال الکتریکی چشم برای ناتوانان جسمی‌حرکتی

http://rehabilitationj.uswr.ac.ir/article-1-1296-fa.html

هدف: هدف این تحقیق طراحی و ساخت و ارزیابی عملکرد یک واسط انسان با کامپیوتر پوشیدنی بوده است که امکان تایپ حروف با حرکات چشم را برای بیان منظور، در افراد ناتوان جسمی‌حرکتی و فاقد قدرت تکلم فراهم می‌آورد. روش بررسی: در این مطالعۀ کاربردی‌توسعه‌ای، عینکِ واسطی طراحی و ساخته شده است که سیگنال الکتریکی چشم را ثبت و به‌صورت بی‌سیم به کامپیوتر منتقل می‌کند. با پردازش زمان حقیقی اطلاعات، امکان ارتباط با صفحه‌کلید مجازی و نیز بازی کامپیوتری فراهم می‌شود. برای ارزیابی عملکرد دستگاه، شش فرد عادی ازلحاظ جسمی‌ذهنی و علاقه‌مند به شرکت در آزمایش، در محدودۀ سنی ۱۶تا۵۰ با نمونه‌گیری ساده انتخاب شدند و در مجموع ۱۰۷۱ کاراکتر را با این دستگاه تایپ کردند. بر‌این‌اساس پارامترهایی نظیر صحت عملکرد سیستم و میزان تایپ محاسبه شد. همچنین امکان‌پذیری استفاده از دستگاه برای افراد ناتوان جسمی‌حرکتی با انجام بازی کامپیوتری روی چهار فرد فلج در چهار اندام بررسی شد. یافته‌ها: طبق نتایج به‌دست‌آمده از آزمایش‌ها، صحت عملکرد سیستم در تشخیص حالت‌‌های حرکتی چشم، در گروه افراد عادی ۹۴/۱ درصد و میزان تایپ ۷/۲۲ کاراکتر در دقیقه محاسبه شد. در انجام حرکت‌‌های چشم برای ارتباط با سیستم، متوسط درصد موفقیت افراد ناتوان جسمی‌حرکتی شرکت‌کننده در آزمایش ۵۸/۷ درصد محاسبه گردید. نتیجه‌گیری: این دستگاه ضمن حفظ کیفیت مطلوب در ثبت و پردازش سیگنال، کوچک (در اندازۀ cm۴/۵×cm۲/۶) و سبک (۱۵ گرم) و کم‌مصرف (توان مصرفی mW ۱۲۳) بوده است، به‌صورت عینکی روی چشم گذاشته می‌شود و امکان کاربرد روزانه را برای کاربر خود فراهم می‌کند. محیط کاربری به‌گونه‌ای طراحی شده است که فرد می‌تواند کلمات را با کمترین تعداد حرکت چشم تایپ کند.

Title: بررسی تأثیر نقطۀ ماشه‌ای فعال عضلۀ تراپزیوس فوقانی روی فعالیت الکترومیوگرافی و حداکثر نیروی این عضله حین انجام اسکاپشن در دو گروه با و بدون نقطۀ ماشه‌ای

http://rehabilitationj.uswr.ac.ir/article-1-1276-fa.html

هدف: نقاط ماشه‌ای می‌توانند منشأ اصلی دردهای تیرکشنده به مناطق اطراف خود باشند. نقاط ماشه‌ای مایوفاشیال پدیدۀ الکتروفیزیولوژیکال هستند که می‌توانند در عملکرد نرمال عضله تداخل ایجاد کنند. هنوز هیچ مطالعه‌ای تأثیرات نقاط ماشه‌ای فعال را بر الگوی حرکت شانه بررسی نکرده است. مطالعات اخیر، تنها دربارۀ افراد سالم انجام گرفته است. ‌این الگوها می‌تواند عامل تعیین‌کننده‌ای در بروز صدمات آینده و منشأ بروز یا تشدید درد‌های گردنی‌شانه‌ای باشد. هدف این تحقیق بررسی میزان فعالیت و زمان تأخیر وارد‌عمل‌شدن عضلۀ تراپزیوس فوقانی در حضور نقطۀ ماشه‌ای فعال، هنگام بالا‌بردن دست در صفحۀ کتف (اسکپشن) بود.  روش بررسی: در یک مطالعۀ مقایسه‌ای موردی‌شاهدی در بهار سال ۱۳۹۱، ۱۷ زن مبتلا به نقطۀ ماشه‌ای فعال عضلۀ تراپزیوس فوقانی با محدوده سنی ۲۶/۷۶ سال و ۱۷ زن سالم با محدوده سنی ۲۶/۱۸ سال، در آزمایشگاه بیومکانیک دانشگاه علوم‌ بهزیستی و توان‌بخشی شرکت داشتند. با استفاده از دستگاه الکترومیوگرافیِ سطحی، میزان فعالیت و زمان شروع فعالیت عضلۀ تراپزیوس فوقانی هنگام حرکت اسکاپشن ثبت و تحلیل شد. یافته‌ها: زمان وارد‌عمل‌شدن عضلۀ تراپزیوس فوقانی نسبت‌به شروع حرکت، در گروه بیمار نسبت‌به گروه سالم به‌صورت معناداری با تأخیر همراه بود (۰/۰۴=P). مقایسۀ میزان حداکثر آمپلی‌تود در افراد مبتلا به نقطۀ ماشه‌ای درمقایسه‌با افراد سالم هنگام حداکثر انقباض ارادی ایزومتریک اسکپشن ۹۰ درجه، در عضلۀ تراپزیوس فوقانی، اختلاف معنادار آماری را به‌صورت کاهش میزان فعالیت در گروه بیمار نشان داد (۰/۰۱=P). نتیجه‌گیری: در حضور نقطۀ ماشه‌ای، الگوی فعالیت عضلات، دستخوش تغییرات می‌شود؛ به‌طوری‌ که نقاط ماشه‌ای با تغییر زمان‌بندی، به الگوی غیرطبیعی حرکت کمربند شانه‌ای منجر می‌شوند. این یافته‌ها می‌تواند در پیشگیری و درمان اختلالات شانه استفاده گردد.
